# Aspirin for the prevention of preeclampsia: A systematic review and meta-analysis of randomized controlled studies

**DOI:** 10.3389/fcvm.2022.936560

**Published:** 2022-11-09

**Authors:** Yixiao Wang, Xiaojun Guo, Nathan Obore, Hongjuan Ding, Chengqian Wu, Hong Yu

**Affiliations:** ^1^Department of Obstetrics and Gynecology, Southeast University Affiliated Zhongda Hospital, Nanjing, China; ^2^Nanjing Maternity and Child Health Care Hospital, Nanjing Maternal and Child Health Institute, Women’s Hospital of Nanjing Medical University, Nanjing, China

**Keywords:** pregnancy, aspirin, preeclampsia, meta-analysis, systematic review

## Abstract

**Background:**

The results of randomized controlled studies on aspirin for the prevention of preeclampsia (PE) are conflicting, and some of the related meta-analyses also have limitations or flaws.

**Data sources:**

A search was conducted on PubMed, Embase, and Cochrane Central Register of Controlled Trials databases, with no time or language restrictions.

**Study eligibility criteria:**

Randomized controlled studies comparing aspirin for the prevention of PE were conducted.

**Methods:**

Systematic reviews were performed according to the Cochrane Manual guidelines. A fixed-effects model or a random-effects model was chosen to calculate pooled relative risks with 95% confidence intervals based on the heterogeneity of the included studies. The study aimed to investigate the effect of aspirin on the development of PE in high-risk and general populations of women. Publication bias was assessed by funnel plots. All included studies were assessed for bias by the Cochrane Manual of Bias Assessment. Subgroup analyses were conducted on the aspirin dose, time of initial aspirin intervention, and the region in which the research was conducted, to explore the effective dose of aspirin and time of initial aspirin intervention and to try to find sources of heterogeneity and publication bias.

**Results:**

A total of 39 articles were included, including 29 studies involving pregnant women at high risk for PE (20,133 patients) and 10 studies involving a general population of pregnant women (18,911 patients). Aspirin reduced the incidence of PE by 28% (RR 0.72, 95% CI 0.62–0.83) in women at high risk for PE. Aspirin reduced the incidence of PE by 30% in the general population (RR 0.70, 95% CI 0.52–0.95), but sensitivity analyses found that aspirin in the general population was not robust. A subgroup analysis showed that an aspirin dose of 75 mg/day (RR 0.50, 95% CI 0.32–0.78) had a better protective effect than other doses. Starting aspirin at 12–16 weeks (RR 0.62, 95% CI 0.53–0.74) of gestation or 17–28 weeks (RR 0.62, 95% CI 0.44–0.89) reduced the incidence of PE by 38% in women at high risk for PE, but the results were more reliable for use at 12–16 weeks. Heterogeneity and publication bias of the included studies may be mainly due to the studies completed in Asia.

**Conclusion:**

Aspirin is recommended to be started at 12–16 weeks of pregnancy in women at high risk for PE. The optimal dose of aspirin to use is 75 mg/d.

**Systematic review registration:**

[www.ClinicalTrials.gov], identifier [CRD42022319984].

## Introduction

Preeclampsia (PE) is a pregnancy-specific disorder that affects approximately 3–5% of pregnant women worldwide, especially in developing countries (1, 2). PE can cause maternal impairment including kidney damage, liver damage, hemolysis, neurology injuries including seizures (eclampsia), stroke, and death (3, 4). Preterm delivery and fetal growth restriction due to PE often have lifelong consequences for the child. These may include cerebral palsy and neurodevelopmental impediment, respiratory disease, hypertension, renal insufficiency, insulin resistance, obesity, cardiovascular disease, and impaired work capacity (5). In 2014, the International Society for the Study of Hypertension in Pregnancy (ISSHP) defined the diagnostic criteria for PE as new-onset hypertension (≥ 140 mmHg systolic or ≥ 90 mmHg diastolic) after 20-week gestation with the coexistence of either proteinuria (≥ 300 mg/day) or other maternal organ dysfunction such as renal insufficiency, liver involvement, neurological or hematological complications, uteroplacental dysfunction, or fetal growth restriction (6).

Currently, the only treatment for prenatal PE is childbirth. The use of drugs such as aspirin, statins, metformin, and proton pump inhibitors for the prevention and treatment of PE remains controversial (7). Aspirin’s effects on inflammation and platelet aggregation may help prevent or treat PE (8), and several randomized controlled trials have been conducted. The ACOG recommends that women with any high-risk factors (PE in previous pregnancies, multiple pregnancies, kidney disease, autoimmune disease, type 1 or 2 diabetes, and chronic hypertension) and multiple moderate risk factors for PE (First pregnancy, maternal age ≥ 35 years, BMI over 30, family history of PE, sociodemographic characteristics, and personal medical history factors) should receive low-dose (81 mg/day) aspirin to prevent PE. This should begin between 12 and 28 weeks of gestation (preferably before 16 weeks of gestation) and continue until delivery (9).

In 2017, a high-quality randomized controlled study found that daily aspirin of 150 mg from 11 to 14 weeks of gestation until 36 weeks of gestation reduced the incidence of preterm PE in women at high risk for PE (10). However, a 2022 study in China found that daily oral administration of 100 mg of aspirin started at 12–20 weeks of gestation to 34 weeks of gestation in high-risk groups of PE did not reduce the incidence of PE. A recent meta-analysis (11) of the effect of aspirin on the occurrence of preterm PE in women at moderate to high risk of PE incorrectly counted the total number of participants as 3,294 (the correct number is 3,269) when extracting data from the study by Subtil et al. (12). While this error may not affect the results of the analysis, it can make the conclusions less rigorous. Another study investigating low-dose aspirin for the prevention of PE did not differentiate between pregnant women at high risk of PE and those in the general population (13). It should be noted that since the general population of women in this study was not screened for PE risk factors, they may have included some women with PE risk factors.

These contradictory or flawed conclusions make clinicians hesitant to use aspirin for the prevention of PE. Therefore, we performed a meta-analysis of published randomized controlled studies on aspirin for the prevention of PE, to obtain more comprehensive conclusions and to try to explain the reasons for the inconsistency of the conclusions of previous studies.

## Methods

This systematic review was conducted as per the Guidelines for Systematic Review and Meta-Analysis (PRISMA) (14). This study only included randomized controlled studies to assess the use of aspirin for the prevention of PE. This study was registered in PROSPERO with the registration number CRD42022319984.

### Eligibility criteria, information sources, and search strategy

We searched PubMed, Embase, and the Cochrane Central Register of Controlled Trials databases for randomized controlled trials related to aspirin and PE. We used MeSH terms and keywords and followed the Harvard Medical School Library’s recommended search strategy for the search as follows. There were no language restrictions, and the databases were searched for the literature published from the time of inception to 9 March 2022 (Supplementary material 1). The search for relevant literature followed the principles of population, intervention, comparator, outcomes, and study design (PICOS). In addition, we also conducted a screening of the included studies for relevant references.

### Study selection

All the articles retrieved from the database were deduplicated by EndNote X9. After browsing the article titles and abstracts, irrelevant articles were excluded and the remaining articles were comprehensively evaluated by two independent reviewers (Yixiao Wang and Xiaojun Guo) to select articles that met the criteria. If there was any disagreement at any time, they were discussed and solved with the corresponding author (Chengqian Wu). The inclusion criteria for this meta-analysis were studies with one group of pregnant women that received aspirin before delivery and the other group that received a placebo or no treatment and studies that documented the incidence of PE. Review articles, editorials, case reports, and conference abstracts were excluded.

### Data extraction

Two authors independently reviewed each study and extracted the following data from the articles: first or corresponding author name, date of publication, the location where the study was conducted, aspirin intervention time and cut-off time, aspirin dose used, the incidence of PE, inclusion and exclusion criteria for the included women, and diagnostic criteria for PE.

### Risk of bias assessment and sensitivity analysis

Publication bias was assessed using funnel plots, and the Cochrane Bias Assessment Manual was used to assess selection bias, performance bias, detection bias, attrition bias, reporting bias, and other biases for all the included studies. We performed sensitivity analyses by excluding individual studies to explore the strength of each included study.

### Subgroup analysis

This study conducted a subgroup analysis to explore the time of initial aspirin intervention and prophylactic aspirin dosage used among PE high-risk groups. In addition, we performed subgroup analyses by region (Africa, America, Asia, and Europe, and involving multiple continents) to explore the sources of heterogeneity and bias.

### Statistical analysis

All data were analyzed by Review Manager software, version 5.3 (Nordic Cochrane Center, Cochrane Collaboration, Copenhagen, Denmark), except for sensitivity analysis which was conducted and plotted using GraphPad Prism 7 software. Forest plots to obtain pooled relative risk (RR) and 95% confidence intervals (95% CI) were drawn. If *I*^2^ < 50% and *P* > 0.10, a fixed-effects model was applied to calculate pooled effect estimates. A random-effects model was applied if *I*^2^ ≥ 50% or *P* ≤ 0.10. Statistical significance was defined as *P* < 0.05.

## Results

### Study selection

**FIGURE 1:**
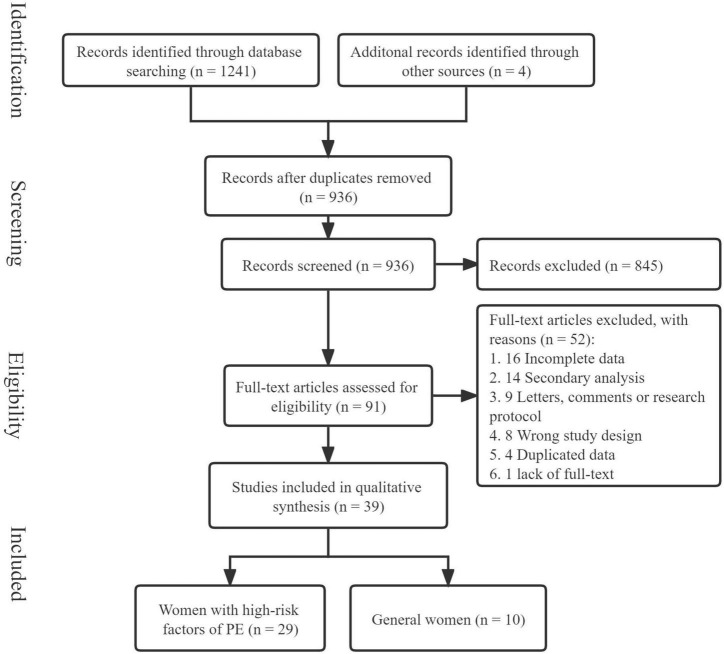
Flowchart of the screening of studies about aspirin and PE.

A total of 1,241 relevant articles from the inception of the databases to 9 March 2022, were retrieved, and 4 articles were included from the references of included articles. After removing duplicates, 936 articles remained, and 845 irrelevant articles were excluded after reading the article titles and abstracts. Of the remaining 91 articles, 52 articles were excluded after careful reading of the full text, and ultimately, our meta-analysis included 39 articles (Figure 1).

### Study characteristics

The main characteristics of the included studies are presented in Table 1. A total of 39 articles were included (12, 15–52), of which 29 studied pregnant women at high risk of PE [a total of 20,133 patients (15, 16, 18–23, 25, 26, 28, 31–40, 42, 43, 45, 47–51)] and 10 studied the general population of pregnant women (a total of 18,911 patients) (12, 17, 24, 27, 29, 30, 41, 44, 46, 52). Among those at high risk for PE, 10,366 women received aspirin prophylaxis before delivery, and 9,767 patients received a placebo or no treatment. Two studies were completed in Africa (19, 22), three in the Americas (20, 23, 38), eight in Asia (15, 26, 33, 34, 37, 42, 45, 47), one in Australia (36), 13 in Europe (16, 18, 25, 28, 31, 32, 35, 39, 43, 48–51), and two covering multiple continents (21, 40). Aspirin was used in doses ranging from 25 to 150 mg/day, and one study used a dose of 0.5 mg/kg/day (48). The initiation of aspirin intervention varied from 11 to 32 weeks of gestation in all but one study that initiated aspirin use at the time of pregnancy diagnosis (34). In the studies involving the general population, 9,416 women received aspirin prophylaxis before delivery, and 9,495 women received a placebo or no treatment. Aspirin was used in doses ranging from 60 to 100 mg/day. The initiation time of aspirin intervention varied from 12 to 32 weeks of gestation, except in one study that started aspirin before pregnancy (27). The inclusion and exclusion criteria for the included studies and their PE diagnostic criteria are presented in Supplementary material 2.

**TABLE 1:** Characteristics of the included studies.

Study	N	Area	Dose of aspirin	Gestational weeks		Aspirin		Control	
						
				Intervention	Stop	PE	Total	PE	Total
**Women with high-risk factors of PE**									
Abdi et al. (15)	86	Asia	80 mg	12–15	36	27	43	38	43
Ayala et al. (16)	350	Europe	100 mg	12–16	Delivery	11	176	22	174
Bower et al. (18)	60	Europe	60 mg	24	-	9	31	12	29
Byaruhanga et al. (19)	230	Africa	75 mg	20–28	-	17	113	23	117
Caritis et al. (20)	2,503	America	60 mg	13–26	Delivery	226	1,254	250	1,249
CLASP (21)	9,309	*	60 mg	12–32	Delivery	313	4,659	352	4,650
Ebrashy et al. (22)	139	Africa	75 mg	14–16	-	26	74	40	65
ECPPA. (23)	970	America	60 mg	12–32	Delivery	32	476	30	494
Grab et al. (25)	43	Europe	100 mg	20	-	3	22	2	21
Gu et al. (26)	1,105	Asia	25–75 mg	12	36	91	821	51	284
Harrington et al. (28)	161	Europe	100 mg	17–23	Delivery	6	58	9	103
Hermida et al. (31)	100	Europe	100 mg	12–16	Delivery	3	50	7	50
Hermida et al. (32)	341	Europe	100 mg	12–16	Delivery	27	174	24	167
Lin et al. (33)	898	Asia	100 mg	12–20	34	78	464	74	434
Liu et al. (34)	98	Asia	100 mg	Diagnosis of pregnancy	Delivery	3	50	10	48
McParland et al. (35)	100	Europe	75 mg	24	40	1	48	10	52
Morris et al. (36)	102	Australia	100 mg	17–19	-	4	52	7	50
Movahed et al. (37)	100	Asia	80 mg	11–14	-	6	50	12	50
Odibo et al. (38)	30	America	81 mg	11–13	36	3	16	3	14
Parazzini (39)	1,042	Europe	50 mg	16–32	Delivery	12	565	9	477
Rolnik et al. (40)	1,620	*	150 mg	11–14	36	56	798	74	822
Schiff et al. (42)	65	Asia	100 mg	28–29	38^+4^	1	34	7	31
Schröcksnadel et al. (43)	41	Europe	80 mg	28–32	37	0	22	6	19
Sun (45)	112	Asia	75 mg	16–22	Delivery	12	54	36	58
Talari et al. (47)	80	Asia	80 mg	12–16	-	1	40	9	40
Vainio et al. (48)	86	Europe	0.5 mg/kg	12–14	-	2	43	10	43
Viinikka et al. (49)	197	Europe	50 mg	12–18	Delivery	9	97	11	100
Villa et al. (50)	121	Europe	100 mg	12–13^+6^	35	8	61	11	60
Wallenburg et al. (51)	44	Europe	60 mg	28	Delivery	0	21	7	23
**General women**									
Bakhti and Vaiman (17)	164	Africa	100 mg	8–10	36	0	82	4	82
Golding (24)	6,049	Africa	100 mg	12–32	-	215	3,023	189	3,026
Haapsamo et al. (27)	107	Europe	75 mg	Before pregnancy	Delivery	4	52	4	55
Hauth et al. (29)	604	America	75 mg	24	Delivery	5	302	17	302
Herabutya et al. (30)	1,348	Asia	80 mg	18–24	-	9	651	19	697
Rotchell et al. (41)	3,641	America	0.5 mg/kg	12–32	Delivery	40	1,819	46	1,822
Sibai et al. (44)	2,985	America	50 mg	13–26	Delivery	69	1,485	94	1,500
Subtil et al. (12)	3,269	Europe	100 mg	14–20	34	28	1,632	26	1,637
Taherian et al. (46)	660	Asia	60 mg	20	Delivery	15	330	33	330
Wang and Li (52)	84	Asia	75 mg	28–30	-	3	40	12	44

*Multiple regions involved; -Not available from study.

### Total pooled effects

**FIGURE 2:**
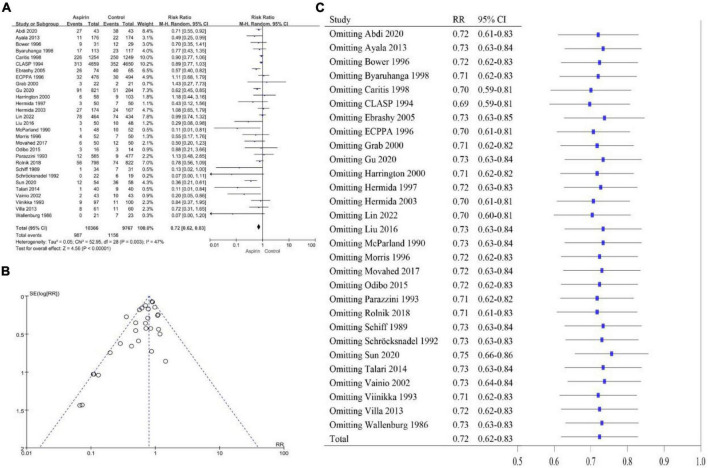
**(A)** Forest plot of studies on the effect of aspirin on the development of PE in women at high risk for PE. **(B)** Funnel plot of the studies on women at high risk for PE. **(C)** Sensitivity analysis of the included studies.

**FIGURE 3:**
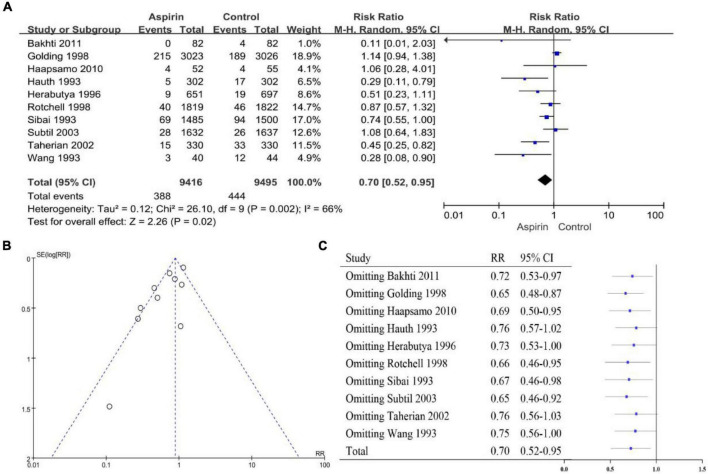
**(A)** Forest plot showing the effect of aspirin on the development of PE in the general population of women. **(B)** Funnel plot showing studies that involved women in the general population. **(C)** Sensitivity analysis of the included studies.

The heterogeneity of the PE high-risk women studies was *I^2^* = 47% (*P* < 0.10), so we chose a random-effects model (Figure 2A). The overall pooled effect showed that aspirin was effective in preventing the onset of PE in high-risk women (RR 0.72, 95% CI 0.62–0.83). Compared with the control group, the use of aspirin reduced the incidence of PE by 28%. The funnel plot showed significant asymmetry, suggesting that there may be some publication bias (Figure 2B). Sensitivity analysis found that omitting any article had no significant effect on RR and 95% CI, and the results were relatively stable (Figure 2C). The heterogeneity of the general population group of studies was *I*^2^ = 66% (*P* < 0.10), so the random-effects model was applied (Figure 3A). The overall pooled effect showed that aspirin was effective in preventing the incidence of PE among women in the general population group (RR 0.70, 95% CI 0.52–0.95). Compared with the control group, the use of aspirin was associated with a 30% reduction in the incidence of PE. The funnel plot showed significant asymmetry (Figure 3B). Sensitivity analysis found that omission of the following studies: Hauth 1993 (RR 0.76, 95% CI 0.57–1.02) (29), Herabutya 1996 (RR 0.73, 95% CI 0.53–1.00) (30), Taherian 2002 (RR 0.76, 95% CI 0.56–1.03) (46), or Wang 1993 (RR 0.75, 95% CI 0.56–1.00) (52), led to significant changes in RR and 95% CI; thus, the findings for this classification were not robust (Figure 3C).

### Bias assessment

**FIGURE 4:**
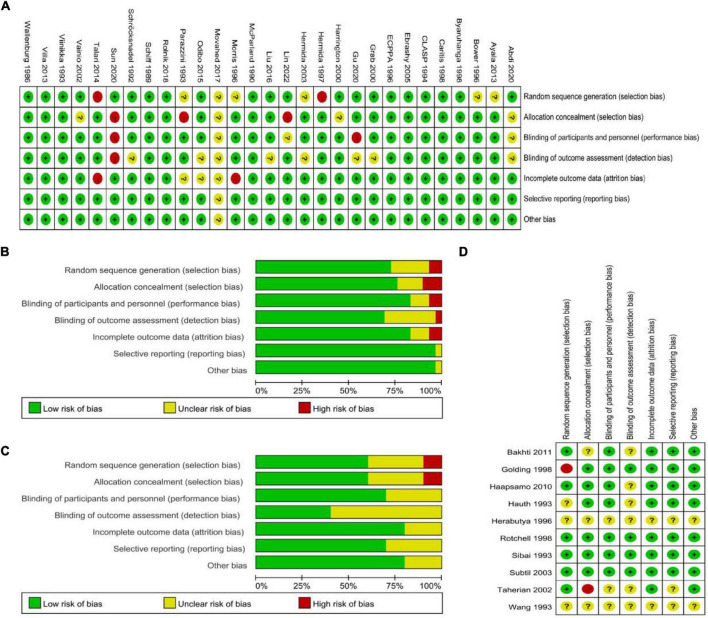
**(A,B)** Bias assessment of studies with PE high-risk women. **(C,D)** Assessment of bias in studies related to the general population of women.

We conducted a bias assessment according to the Cochrane Risk of Bias template. In the PE high-risk women group, two studies had a high risk of random sequence generation (31, 47) and six studies had an unclear risk (16, 18, 32, 36, 37, 39); three studies had a high risk of allocation concealment (33, 39, 45) and four studies had an unclear risk (15, 28, 37, 48); two studies had a high risk of performance bias (26, 45) and three studies had an unclear risk (15, 33, 37); one study had a high risk of detection bias (45) and eight studies had an unclear risk (15, 25, 26, 32, 34, 37, 38, 43); two studies had a high risk of attrition bias (36, 47) and three studies had an unclear risk (37–39); and one study had an unclear reporting bias (37) and one study had unclear other biases (37) (Figures 4A,B). Among the studies on the general population of women, one study had a high risk of random sequence generation (24) and three studies had an unclear risk (29, 30, 52); one study had a high risk of allocation concealment (47) and three studies had an unclear risk (17, 30, 52); three studies had an unclear performance bias (30, 46, 52); and six studies had an unclear detection bias (17, 27, 29, 30, 46, 52). Attrition bias was unclear in two studies (30, 52). In three studies, reporting bias was not clear (30, 46, 52). Two studies had unclear other biases (30, 52) (Figures 4C,D).

### Subgroup analysis

#### Dosage of aspirin

**FIGURE 5:**
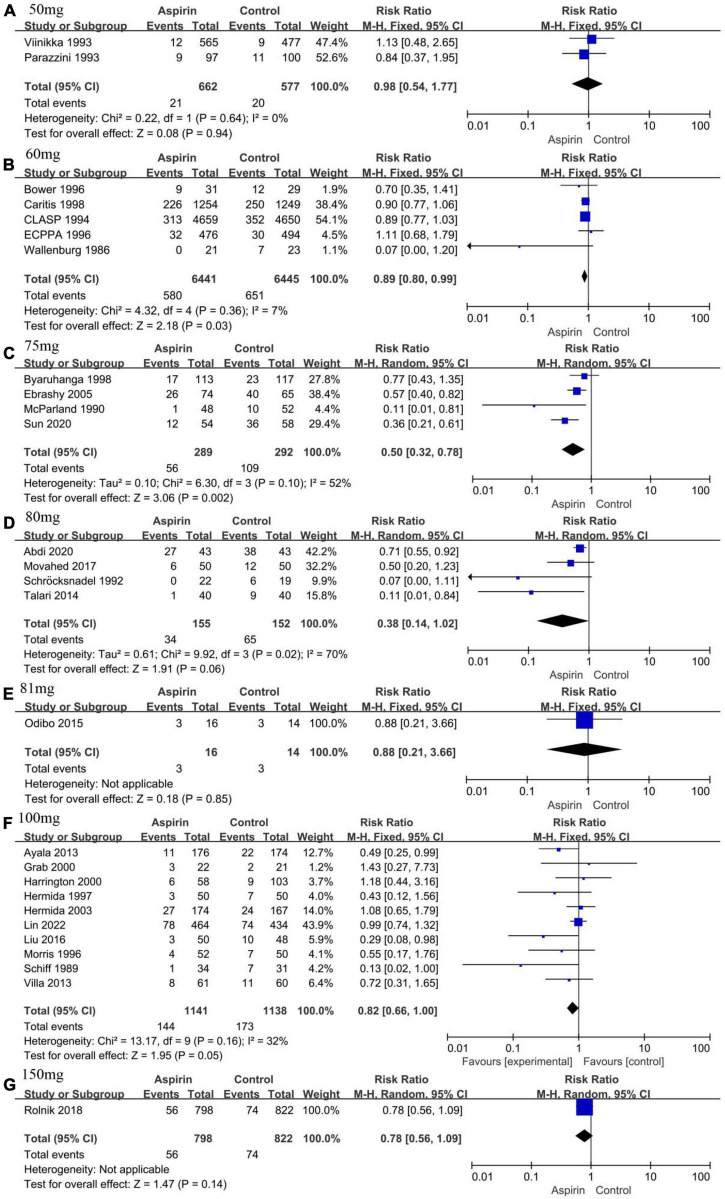
Forest plot showing subgroup analysis according to aspirin dose. **(A)** 50 mg/day. **(B)** 60 mg/day. **(C)** 75 mg/day. **(D)** 80 mg/day. **(E)** 81 mg/day. **(F)** 100 mg/day. **(G)** 150 mg/day.

The ACOG recommends that pregnant women with any risk factors for PE should receive low-dose aspirin (81 mg/day) to prevent the development of PE (9). We performed subgroup analyses based on aspirin dose because different doses of aspirin were used in each study. The groups were divided as follows: 50, 60, 75, 80, 81, 100, and 150 mg/day. Two studies were not included in the subgroup analysis because they used 0.5 mg/kg/day (26) and 25–75 mg/day (48) of aspirin, respectively. The incidence of PE was reduced by 11 and 50% with 60 mg/day (RR 0.89, 95% CI 0.80–0.99, Figure 5B) and 75 mg/day (RR 0.50, 95% CI 0.32–0.78, Figure 5C) of aspirin, respectively. In addition, sensitivity analysis showed that the pooled results of a dosage of 60 mg/d of aspirin were not reliable (Supplementary Figure 1B). The risk of PE was not reduced when other dosages of aspirin were used (Figures 5A,D–G). The funnel plot was relatively symmetrical in all groups except for the 100 mg/d aspirin group, where it was asymmetrical (Supplementary Figure 2).

#### Timing of initiation of aspirin interventions

**FIGURE 6:**
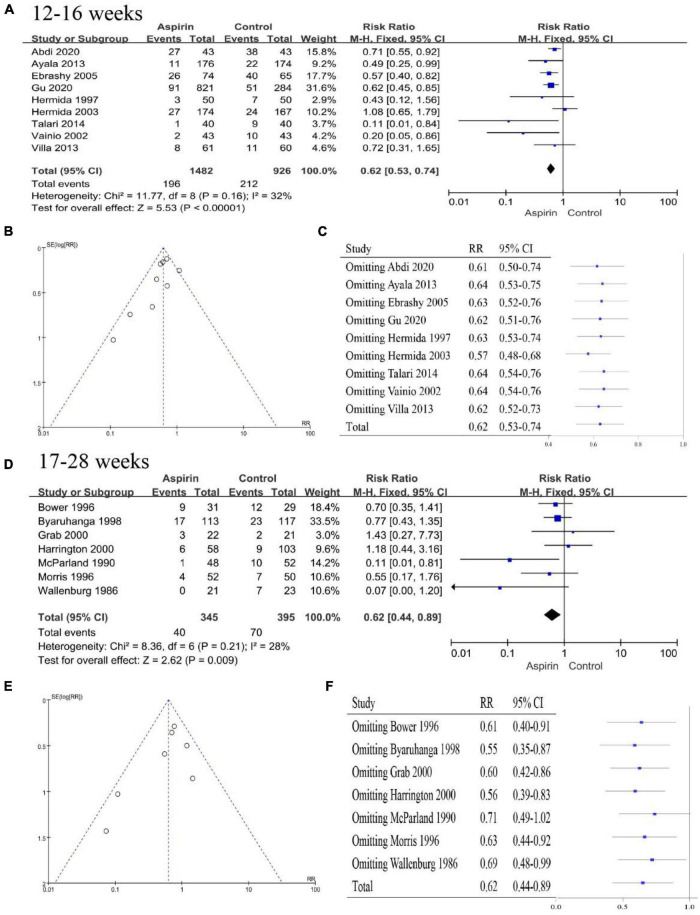
Subgroup analysis by time of initial aspirin intervention. **(A)** Forest plot showing the intervention at 12–16 weeks. **(B)** Funnel plot showing the intervention at 12–16 weeks. **(C)** Sensitivity analysis of the intervention at 12–16 weeks. **(D)** Forest plot for the intervention at 17–28 weeks. **(E)** Funnel plot showing the intervention at 17–28 weeks. **(F)** Sensitivity analysis of the intervention at 17–28 weeks.

The aspirin intervention recommended by the ACOG begins between 12 and 28 weeks of gestation (preferably before 16 weeks of gestation) and continues into labor (9). However, the timing of initiation of aspirin interventions in the studies was not always as recommended by the guidelines. Therefore, we performed a subgroup analysis of the timing of the initiation of aspirin intervention. All studies were divided into 12–16 weeks of gestation (15, 16, 22, 26, 31, 32, 47, 48, 50) and 17–28 weeks of gestation (18, 19, 25, 28, 35, 36, 51) according to the time of initiation of the intervention. The pooled effect of aspirin intervention at 12–16-weeks gestation showed a 38% reduction in the risk of PE (RR 0.62, 95% CI 0.53–0.74, Figure 6A), with essential symmetry on both sides of the funnel chart (Figure 6B). Omission of any of the studies did not cause the conclusion to change, and thus, this conclusion was robust (Figure 6C). The pooled effect of the aspirin intervention from 17 to 28 weeks of gestation showed a 38% reduction in the risk of PE (RR 0.62, 95% CI 0.44–0.89) (Figure 6D), with a largely symmetric funnel plot (Figure 6E). Notably, the total pooled effect size changed significantly when McParland 1990 (35) was omitted (RR 0.71, 95% CI 0.49–1.02, Figure 6F). Therefore, the intervention at 17–28 weeks of gestation was less robust or effective than the intervention at 12–16 weeks of gestation. Aspirin intervention should be started at 12–16 weeks of gestation if possible.

#### Continent where the study was conducted

**FIGURE 7:**
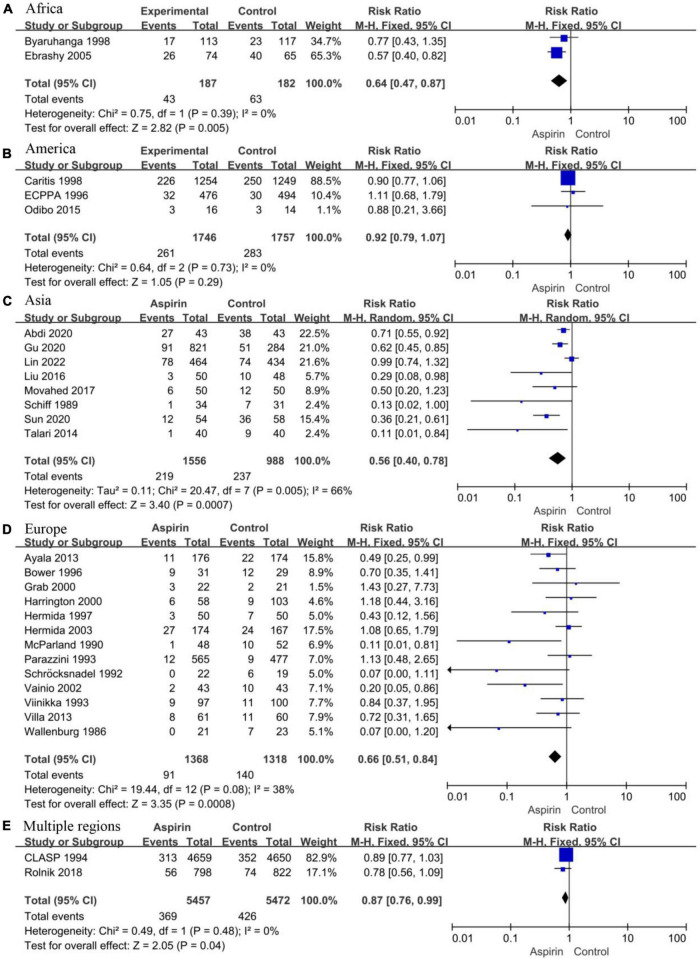
Forest plot showing subgroup analysis by study region. **(A)** Africa. **(B)** America. **(C)** Asia. **(D)** Europe. **(E)** Multiple regions.

Considering the apparent asymmetry of the funnel plot shown in Figure 2A, *I*^2^ = 47%, *P* < 0.10, and Figure 2B, we conducted a subgroup analysis by continent for all the studies to explore possible sources of heterogeneity and publication bias. The studies were divided into an African group (*N* = 2, *I*^2^ = 0%, *P* = 0.39, Figure 7A), an American group (*N* = 3, *I*^2^ = 0%, *P* = 0.73, Figure 7B), an Asian group (*N* = 8, *I*^2^ = 66%, *P* < 0.10, Figure 7C), an European group (*N* = 13, *I*^2^ = 38%, *P* = 0.08, Figure 7D), and a multi-regional group (*N* = 2, *I*^2^ = 0%, *P* = 0.48, Figure 7E). Except for the Asian subgroup, which shows significant asymmetry in the funnel plot, studies conducted on all continents show no significant asymmetry (Supplementary Figure 3). In addition, the results of sensitivity analysis showed that the results were stable in all groups (Supplementary Figure 4). Therefore, we believe that the main reason for the high heterogeneity and publication bias lies in studies conducted in Asia.

## Discussion

We analyzed some previous meta-analyses that examined the use of aspirin for the prevention of PE and identified certain issues with their research conclusions. A meta-analysis published in 2021 did not differentiate between high-risk groups and the general population of women. The study concluded that starting low-dose aspirin before 20 weeks of gestation can significantly reduce the incidence of PE (13).

Our study shows that aspirin is effective in preventing the occurrence of PE in high-risk groups. In the general population of women not screened for high-risk factors for PE, the results of sensitivity analysis showed that the effect of aspirin was unstable, and this is consistent with the results of another meta-analysis examining the protective effect of aspirin on PE in low-risk nulliparous women (RR 0.70, 95% CI 0.47–1.05, *P* = 0.08) (53).

Aspirin belongs to the family of non-steroidal anti-inflammatory drugs, and its analgesic, antipyretic, and anti-inflammatory effects are due to the inactivation of cyclooxygenase-1 (COX-1) and cyclooxygenase-2 (COX-2), inhibiting the production of prostaglandins and thromboxane (54, 55). This reduced thromboxane can also inhibit platelet aggregation, resulting in an antithrombotic effect. There is increasing evidence that suboptimal invasion of trophoblasts leads to an imbalance of angiogenic and antiangiogenic proteins, ultimately leading to extensive inflammation and endothelial damage, increased platelet aggregation, and thrombotic events in placental infarction (56). Due to the possible mechanism of aspirin in preventing PE, we excluded the general population of women who were not screened for high-risk factors for PE in subsequent analyses and focused on the protective effect of aspirin in PE high-risk groups.

Furthermore, we conducted a subgroup analysis of the differences in the timing and dosage of aspirin intervention. The dose of aspirin recommended by the ACOG is 81 mg/day; however, the conventional aspirin dose in some countries is not 81 mg, for example, the common aspirin doses in China are 50 mg, 75 mg, and 100 mg. The use of a 75 mg/day aspirin dose significantly reduced the incidence of PE (RR 0.50, 95% CI 0.32–0.78). Concerning the timing of aspirin intervention, we recommend intervention in high-risk women at 12–16 weeks of gestation given that placenta implantation is essentially completed within 14–18 weeks of gestation (57).

Considering the significant heterogeneity and potential publication bias of the 29 included studies, we performed subgroup analyses by study region. The results showed that, except for the eight studies conducted in Asia, which had significant heterogeneity (*I^2^* = 66%, *P* < 0.10), the heterogeneity of studies conducted in other regions was not significant. In the funnel diagram of each region, only Asia showed obvious asymmetry. We speculate that differences in ethnicities, regions, and countries in Asia, the interaction between internal (including genetic factors, metabolic factors, and pharmacokinetics) and external factors (including environmental, cultural differences, and dietary habits), as well as different definitions of high-risk women, etc., may contribute to the heterogeneity (58, 59).

Our study has some limitations. It was not possible to define specific risk factors for PE, for example, some studies used umbilical artery Doppler to identify high-risk groups and some studies included pregnant women diagnosed with PE in a previous pregnancy. The inability to differentiate between risk factors may affect the reliability of the conclusions. Notably, postpartum PE was not described or discussed in any of the studies, which may lead to errors in the statistics of the incidence of PE. After uniform criteria are obtained for the diagnosis of postpartum PE, studies on the prevention and treatment of postpartum PE with aspirin should be conducted.

In addition, we did not perform regression analysis when exploring the source of heterogeneity. This was because some studies included baseline characteristics of subjects who were lost to follow-up or dropped out, and this could pose unknown risks to the regression analysis. Moreover, only some of the randomized controlled studies accounted for patient compliance, and this may have potential implications for the conclusions of the studies.

Previous studies have shown that aspirin reduces the risk of complications such as preterm birth, perinatal death, and intrauterine growth retardation without increasing the risk of hemorrhage. So, we did not analyze other complications or side effects associated with aspirin use.

## Conclusion

Aspirin is currently widely accepted for the prevention and treatment of PE; however, relevant research conclusions are inconsistent. Multicenter randomized controlled placebo studies involving various ethnicities and regions are needed. This meta-analysis finds that aspirin is only suitable for the prevention of PE among high-risk groups of women and its effect is better when initiated at 12–16 weeks of pregnancy at the recommended dose of 75 mg/day.

## Data availability statement

The original contributions presented in this study are included in the article/Supplementary material, further inquiries can be directed to the corresponding author/s.

## Author contributions

YW and XG were responsible for the study design, data extraction and analysis, and article writing. NO was responsible for the research design and direction and language polish. HD was responsible for the study design and direction. CW and HY were responsible for the research design and revision, manuscript review, and guidance. All authors contributed to the article and approved the submitted version.
