# Supplementary material for: Aspirin for the prevention of preeclampsia: A systematic review and meta-analysis of randomized controlled studies
Source: Front Cardiovasc Med. 2022 Nov 9;9:936560. doi: 10.3389/fcvm.2022.936560 (PMC9682183; doi:10.3389/fcvm.2022.936560)
Supplement: Supplementary file 1 [file Data_Sheet_1.doc]

| **Supplementary material 1.** Database search record |
| --- |
| **Pubmed**  #1 "Preeclampsia"[Mesh]  #2 "Aspirin"[Mesh]  #3 (clinical[tiab] AND trial[tiab]) OR "clinical trials as topic"[mesh] OR "clinical trial"[pt] OR random*[tiab] OR "random allocation"[mesh] OR "therapeutic use"[sh]  #4 ((#1) AND (#2)) AND (#3)  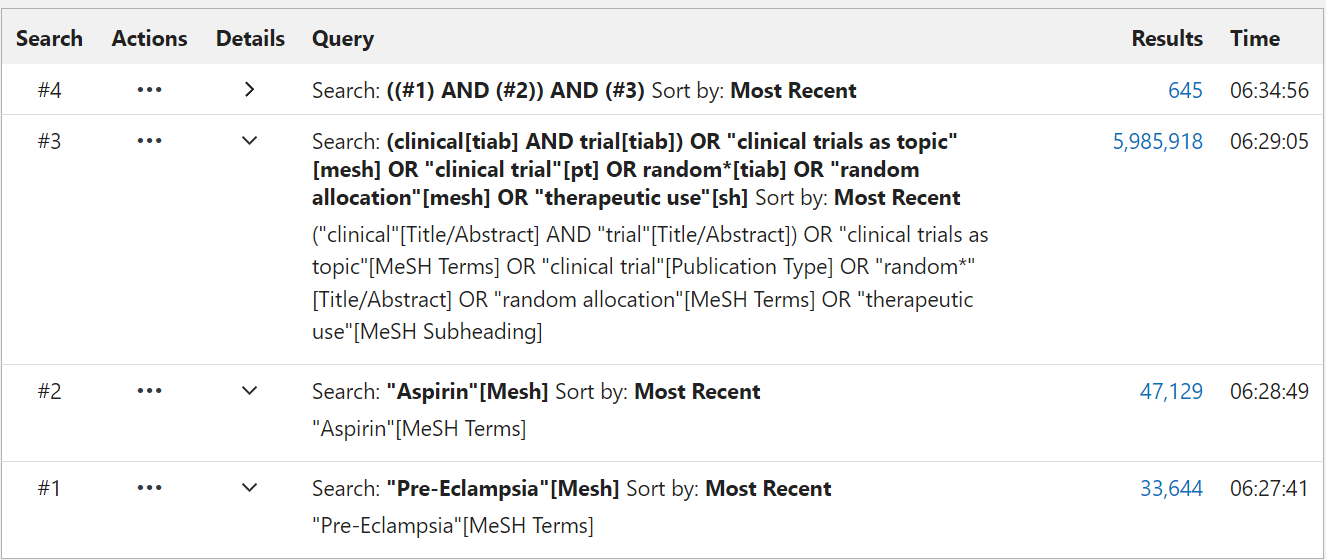 |
| **Embase**  #1 MeSH descriptor: [Aspirin] explode all trees 6168  #2 MeSH descriptor: [Pre-Eclampsia] explode all trees 988  #3 #1 AND #2 in Trials 140  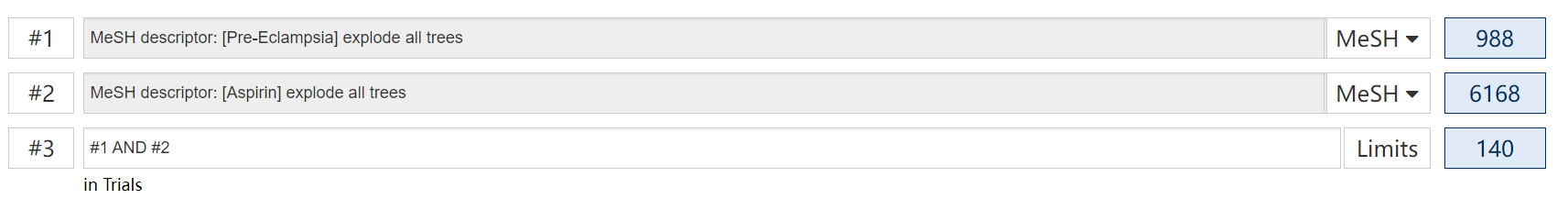 |
| **Cochrane Central Register of Controlled Trials databases**  #5 #3 AND #5 456  #4 'randomized controlled trial':ab,ti OR 'randomized':ab,ti OR 'placebo':ab,ti OR 'rct':ab,ti 1,047,350  #3 #1 AND #2 2,427  #2 aspirin 125,090  #1 'preeclampsia'/exp OR preeclampsia 75,302  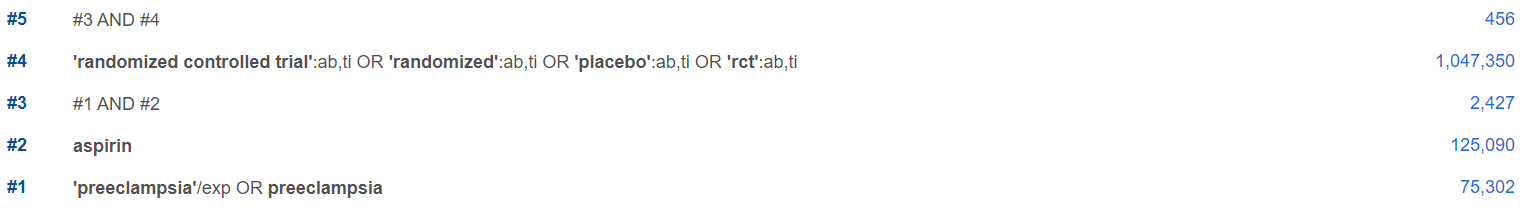 |
