# Supplementary material for: Aspirin for the prevention of preeclampsia: A systematic review and meta-analysis of randomized controlled studies
Source: Front Cardiovasc Med. 2022 Nov 9;9:936560. doi: 10.3389/fcvm.2022.936560 (PMC9682183; doi:10.3389/fcvm.2022.936560)
Supplement: Supplementary file 2 [file Data_Sheet_2.docx]

| **Supplementary material 2.** The inclusion and exclusion criteria for the included studies and their PE diagnostic criteria | | | | | |
| --- | --- | --- | --- | --- | --- |
| Study | Inclusion criteria | | Exclusion criteria | | Diagnostic criteria for PE |
|  |  |  |  |  |  |
| Women with high-risk factors of PE | |  | |  |  |
| Abdi 2020^[14]^ | Pregnant women with gestational age of 12 to 15 weeks and a history of PE in previous pregnancies (at least one previous pregnancy). | | Multiple gestations, gestational diabetes mellitus, chronic medical diseases (eg, hypertension or diabetes mellitus), smoking, coagulation disorders, abnormal uterine artery Doppler at ultrasound screening, abnormal serum level of pregnancy-associated plasma protein A (PAPP-A) at the first-trimester screening, allergy to aspirin, and unwillingness to participate in the research. | | - |
| Ayala 2013^[15]^ | Pregnant women with high risk factors for gestational hypertension or PE, including: familial or personal history of either gestational hypertension or PE; chronic hypertension; cardiovascular, endocrine, bleeding, or metabolic disease; personal history of spontaneous abortion;multiple pregnancy; obesity; and adolescent or middleaged nulliparous pregnancy (<18 or >35 yrs). Additional inclusion criteria for this trial were gestational age ≤16 weeks at randomization and maternal age ≥18 years. | | Multiple pregnancy, chronic hypertension or any other condition requiring the use of BP-lowering medication, cardiovascular disorders (unstable angina pectoris, heart failure, life-threatening arrhythmia, atrial fibrillation, kidney failure, and grade III–IV retinopathy), chronic liver disease, any disease requiring the use of antiinflammatory medication, diabetes or any other endocrine disease such as hyperthyroidism, history of drug/alcohol abuse, night/shiftwork employment, acquired immunodeficiency syndrome (AIDS), intolerance to ABPM, and inability to communicate and comply with all of the study requirements. | | PE defined by a systolic blood pressure >140 mmHg and diastolic blood pressure >90 mmHg with >300 mg per day proteinuria. |
| Bower 1996^[17]^ | Doppler ultrasound screening of both uterine arteries was performed in two stages at 18 to 22 weeks and 24 weeks of gestation as previously described. Women with persistently abnormal waveforms were asked to enter the CLASP trial under the category of prophylactic entry. | | - | | PE was defined as pregnancy-induced hypertension with significant proteinuria (hypertension was defined as two recordings of diastolic blood pressure at or above 90 mmHg, 4 h apart, significant proteinuria was considered to be at least 300 mg/24 h or at least ++ on dipstick testing). |
| Byaruhanga 1998^[18]^ | Pregnant women at 20-28 weeks gestation with the following risk factors: previous history of pregnancy-induced hypertension, PE, especially that occurring before 32 weeks of gestation, or eclampsia; and pre-existing chronic hypertension. | | History of hypersensitivity to aspirin;use of non-steroidal anti-inflammatory drugs; history of peptic ulcer disease;bleeding disorders;history of chronic pulmonary disease; or development of PE prior to entry of trial. | | PE was defined as hypertension with proteinuria developing after 20 weeks of gestation in the antepartum, intrapartum, or post-partum period in a previously normotensive woman (Significant proteinuria was defined using dipsticks as greater than 1+, or presence of 300 mg of protein or more per litre). |
| Caritis 1998^[19]^ | One of four high-risk groups: women with pregestational, insulin-treated diabetes mellitus, women with chronic hypertension, women with multifetal gestations, and women who had had PE in a previous pregnancy. | | Women with multifetal gestations were ineligible for the study if they also had diabetes mellitus, chronic hypertension, or proteinuria, as were women with a history of PE and current proteinuria. | | PE was defined in the women who did not have hypertension or proteinuria at base line as the development of hypertension plus one of the following: proteinuria, thrombocytopenia, or pulmonary edema. Hypertension was defined as either a systolic blood pressure 140 mmHg or a diastolic blood pressure 90 mmHg on two occasions at least four hours apart. Proteinuria was defined as excretion of 300 mg of protein in a 24-hour urine collection, or two dipstick-test results of (100 mg per deciliter), the values recorded at least 4 hours apart, with no evidence of urinary tract infection. |
| CLASP 1994^[20]^ | A history of PE or IUGR in a previous pregnancy, chronic hypertension, renal disease, or other risk factors, such as maternal age, or multiple pregnancy. family history. | | Contraindications included an increased risk of bleeding, asthma, allergy to aspirin, or a high likelihood of immediate delivery. | | The study outcome of proteinuric PE required the development of hypertension and proteinuria after randomisation. For those with baseline diastolic pressure below 90 mmHg, hypertension was defined as a rise of at least 25 mmHg, to 90 mmHg or higher. For those with an initial diastolic pressure of 90 mmHg or above, an increment of at least 15 mmHg was required. Proteinuria was defined as the appearance after randomisation of at least 1+ on protein stick-testing during pregnancy, without evidence of urinary tract infection. |
| Ebrashy 2005^[21]^ | Gestational age between 14 and 16 weeks, and a high risk factor for PE or IUGR, such as previous history of the disease, essential hypertension, positive family history or underlying vascular disorder, maternal age <20 years or >40 years, and gestational diabetes mellitus. Patients with abnormal Doppler results (unilateral or bilateral diastolic incisions and a uterine artery resistance index above the 90th percentile for age) were invited to the trial. | | Cases with a known history of salicylate allergy, present or past peptic ulcer, past use of prostaglandin inhibitors within 10 days before the beginning of the study, as well as cases with other medical disorders such as chronic renal disorders, thyroid diseases, and hepatic and cardiac disorders. | | PE was defined as development of hypertension (140/90 mmHg or more) plus proteinuria (>300 mg protein in 24-hour urine sample). |
| ECPPA 1996^[22]^ | Pregnant women at 12 to 32 weeks of gestation with sufficient risk of PE or its sequelae, including chronic hypertension detected before or during pregnancy, primigravidity (especially with other risk factors, such as young or old age), diabetes, renal disease, a history of PE or intrauterine growth retardation (IUGR) in a previous pregnancy or evidence of their presence in the current pregnancy. | | Contraindications included an increased risk of bleeding, asthma, allergy to aspirin, gastric ulcer, and placenta pruevia. | | Proteinuric PE required the development of hypertension plus the detection of protein in the urine after randomisation. Hypertension was defined for those with baseline diastolic pressure below 90 mmHg as a rise of at least 25 mmHg to 90 mmHg or higher; for those with initial diastolic pressure of 90 mmHg or above, an increment of 15 mmHg was required. |
| Grab 2000^[24]^ | Singleton pregnancies of less than 20 gestational weeks with early intrauterine growth restriction, impaired uteroplacental blood, chronic hypertension or history of stillbirth, growth restriction or PE. | | Diabetes mellitus, pre-existing proteinuric hypertension or fetal malformations or chromosome abnormalities. | | PE defined as proteinuric hypertension. |
| Gu 2020^[25]^ | Pregnant women at high-risk for PE, with one or more high-risk factors or with 2 or more medium-risk factors. High-risk factors included history of PE or gestational hypertension, chronic hypertension, multiple pregnancies, kidney disease, type 1 or type 2 diabetes and autoimmune diseases such as systemic lupus erythematosus and antiphos- pholipid syndrome. Medium risk factors included primipara, age ≥35 years, BMI ≥30 kg/m^2^, family history of PE, poor social and economic status, personal history (polycystic ovary syndrome, low birth weight infant or infant younger than gestational age, >10 years since the previous pregnancy and history of an adverse pregnancy outcome). | | History of chronic illness (excluding hypertension, kidney disease, chronic diabetes mellitus without hypertension and nephropathy); planned delivery at another hospital; severe fetal malformations; anticoagulation therapy during pregnancy or participant in another study on PE prevention. The contraindications for aspirin use are as follows: allergies to aspirin, other salicylates or other ingredients in prescribed drugs; history of asthma caused by salicylates or salicylic acid hydrates or non-steroidal anti-inflammatory drugs; active peptic ulcer; hemorrhagic constitution; severe renal failure; severe liver failure; severe heart failure or in combination with methotrexate (15 mg/week or more). | | PE is defined by the combination of a high blood pressure (the systolic blood pressure should be >140 mmHg and/or the diastolic blood pressure should be >90 mmHg on at least two occasions four hours apart developing after 20 weeks of gestation in previously normotensive women) and proteinuria (>300 mg in 24 h or two readings of at least ++ on dipstick analysis of midstream or catheter urine specimens if no 24 -h collection is available). |
| Harrington 2000^[27]^ | Presenting for a routine anomaly scan between the 19th and 21st week of pregnancy, in addition to the following conditions: otherwise healthy mothers with a normal mix of primigravidae, multigravidae, and different ethnic groups. | | Multiple pregnancy, a history of bleeding in the current pregnancy, asthma requiring treatment, known diabetes, chronic hypertension, and known allergy to aspirin. | | The development of PE was based on the definitions given by the CLASP study: for those with baseline diastolic pressure below 90 mmHg, hypertension was defined as a rise of at least 25 mmHg, to 90 mmHg or higher , while for those with an initial diastolic pressure of 90 mmHg or above, an increment of at least 15 mmHg was required, proteinuria was defined as the appearance of at least ≥1 on protein stick-testing during pregnancy without evidence of urinary tract infection. |
| Hermida 1997^[30]^ | Absence of any condition requiring the use of antihypertensive medication, maternal age (18 to 40 years), and gestational age (<16 weeks). | | Multiple pregnancy, chronic hypertension, chronic liver disease, any disease requiring the use of anti-inflammatory medication, diabetes or any other endocrine disease such as hyperthyroidism, as well as intolerance to the use of an ambulatory BP monitor. | | PE defined as gestational hypertension and proteinuria, above 300 mg/24 h, with or without edema. |
| Hermida 2003^[31]^ | Absence of any condition requiring the use on antihypertensive medication, maternal age (18 to 40 years) and gestational age (<17 weeks). | | Multiple pregnancy, chronic hypertension, chronic liver disease, any disease requiring the use of antiinflammatory medication, diabetes or any other endocrine disease such as hyperthyroidism, and intolerance to ABPM device. | | - |
| Lin 2022^[32]^ | Age ≥18 and <55 years;singleton pregnancy; live fetus at the gestational age of 12 to 20 weeks;definition of high risk of developing PE: (a) at least 1 high risk factor, namely, history of PE, diabetes mellitus (type 1 or 2), or chronic hypertension; or (b) at least 2 of the following intermediate risk factors, including obesity (prepregnancy body mass index [pre-BMI] ≥ 28 kg/m), advanced maternal age (≥ 35 years), family history of PE (mother or/and sister) or nulliparity; ability to undergo all procedures listed in the protocol; a written informed consent for participation in the study. | | Allergy to aspirin; asthma; peptic ulcers; no tolerance to this study because of severe heart, liver, or renal disease; autoimmune diseases; mental disorders; history of alcohol or drug abuse within 6 months; in-vitro fertilization; previous registration in another drug trial within the previous 3 months. | | PE was diagnosed with SBP ≥140 mmHg or DBP ≥90 mmHg on at least two occasions 4 hours apart, developing after 20 weeks of gestation with previously normal blood pressure (SBP <140 mmHg and DBP < 90 mmHg), and accompanied by proteinuria. Any of the following criteria were used for diagnoses of proteinuria: protein in urine is ≥ 300mg/24 hours of urine collection (or can be extrapolated from a timed collection), protein/creatinine ratio is ≥ 0. 3mg/dL, dipstick reading 2+ (used only if other quantitative methods are not available), and new-onset vital organ damage. |
| Liu 2016^[33]^ | 1. Age ≥18; Single birth; No abortion history. Have the following risk factors: age ≥40, BMI ≥35 kg/m^2^, PE family history, multiple pregnancy history, previous hypertensive disease, chronic nephrosis, autoimmune disease like systemic lupus erythematosus; systemic lupus erythematosus and antiphospholipid syndrome, type 1 or type 2 diabetes, chronic hypertension well controlled. | | Coagulation disorder; Need oral intake of anticoagulants like Warfarin or vein application of heparin; Have hypertension before pregnancy and fail to control up to standard with drug;Being allergic to aspirin, low compliance. | | - |
| McParland 1990^[34]^ | Patients underwent doppler ultrasound examination of the utero placental circulation as previously described. Patients with abnormal waveforms had repeat examinations at 24 weeks’ gestation, and if the wave forms were still abnormal they were invited to take part in the trial. | | Known aspirin allergy, maternal diabetes mellitus, bleeding disorders, peptic ulceration, and systemic lupus erythematosus. | | Hypertension in pregnancy (Diastolic blood pressure of ≥110 mmHg on anyone occasion or A diastolic blood pressure of ≥90 mmHg on two or more consecutive occasions ≥4 hours apart) with Proteinuria (One 24-hour urine collection with a total protein excretion of ≥300 mg per 24 hours or Two "clean-catch-midstream" or catheter specimens of urine collected ≥4 hours apart with:1. 1 gm albumin per liter or 2+ more on reagent strip or sulfosalicylic acid "cold" test or 2. 0. 3 gm albumin per liter or 1+ on reagent strip if specific gravity <1. 030 and pH <8) |
| Morris 1996^[35]^ | At 17-19 weeks of gestation, if abnormal uterine placental resistance is found, the women are required by one of the three clinicians (JMM, RAF or DAE) to participate in a randomized, double-blind, placebo-controlled trial. | | Pregnancy as less than 17 weeks or more than 19 weeks and 6 days. | | Pregnancy-induced hypertension was defined as antenatal blood pressure (BP) exceeding 140/90 mmHg, with a rise in diastolic pressure of at least 15 mmHg on at least two occasions 6 hours apart, not including those readings taken in labor. PE was defined as pregnancy-induced hypertension plus proteinuria (l+ or more on dipstick testing on at least two occasions 6 hours apart) or hyperuricemia. |
| Movahed 2017^[36]^ | All singleton pregnant women at 11-14 weeks of gestation were evaluated by uterine artery Doppler ultrasound and in the case of abnormal findings, were divided into two groups of case and control. | | - | | - |
| Odibo 2015^[37]^ | Singleton pregnancy undergoing ultrasound examination at 11 + 0 to 13 + 6 weeks and deemed to be at high risk for PE by the criteria:Chronic hypertension 4, History of prior PE, Diabetes mellitus, obesity (BMI > 30), bilateral uterine artery notches, Low pregnancy-associated plasma protein-A. | | Pregnancies with multiple gestation, fetal aneuploidy, major fetal structural anomaly and bleeding disorder, and women with allergy to aspirin or already on aspirin or heparin. | | Blood pressure of 140 mmHg systolic or higher or 90 mmHg diastolic or higher that occurs after 20 weeks of gestation in a woman with previously normal blood pressure. Proteinuria, defined as urinary excretion of 0. 3g protein or higher in a 24-hour urine specimen. |
| Parazzini 1993^[38]^ | Pregnant women (between 16 to 32 weeks of gestation) who satisfied one or more of the following criteria.   For those treated prophylactically, the criterias were age under 18 or over 40 years; Mild or moderate chronic hypertension (diastolic pressure between 90 and 110 mmHg); nephropathy with normal renal function and normal blood pressure; History of PIH with or without proteinuria after week 32 of a previous pregnancy;  History of IUGR (baby below 10th centile); and current twin pregnancy.  For those who received aspirin therapeutically, the criteria were PIH (diastolic pressure between 90 and 110mmHg) in this pregnancy or early signs of IUGR (fetal abdominal circumference ≥ 2SD below the mean for gestational age). | | Women with a history of chronic disease (except hypertension, renal disease and chronic diabetes without hypertension and nephropathy), allergy to aspirin, or documented fetal malformations. | | - |
| Rolnik 2018^[39]^ | An age of 18 years or more, singleton pregnancy, live fetus at the time that scanning was performed at 11 to 13 weeks of gestation, and a high risk (>1 in 100) for preterm PE according to the screening algorithm. | | Unconscious or severely ill status, learning difficulties or serious mental illness, major fetal abnormality identified at the time that scanning was performed at 11 to 13 weeks of gestation, regular treatment with aspirin within 28 days before screening, bleeding disorder such as von Willebrand’s disease, peptic ulceration, hypersensitivity to aspirin, long-term use of nonsteroidal anti-inflammatory medication, and participation in another drug trial within 28 days before screening. | | The systolic blood pressure should be 140 mmHg or more and/or the diastolic blood pressure should be 90 mmHg or more on at least two occasions four hours apart developing after 20 weeks of gestation in previously normotensive women. Hypertension should be accompanied by proteinuria of 300 mg or more in 24 hours or two readings of at least ++ on dipstick analysis of midstream or catheter urine specimens if no 24-hour collection is available. In PE superimposed on chronic hypertension significant proteinuria (as defined above) should develop after 20 weeks of gestation in women with known chronic hypertension (history of hypertension before conception or the presence of hypertension at the booking visit before 20 weeks’ gestation in the absence of trophoblastic disease). |
| Schiff 1989^[41]^ | Pregnant women have at least one of these risk factors:undelivered pregnancy, twin pregnancy, and a history of PE toxemia.  Then，those who have undergone a positive selective flip test at 28 or 29 weeks of gestation. | | History of chronic (primary) hypertension, long-term use of nsaids or use of such drugs in the past 6 weeks, discovery of PIH or proteinuria prior to screening. | | Proteinuric pregnancy-induced hypertension, or PE toxemia, was defined as hypertension(systolic blood pressure in excess of 140 mmHg, diastolic blood pressure in excess of 90 mmHg, or both, when measured on at least two occasions within 24 hours of each other) accompanied by proteinuria (>1g in 24 hours). |
| Schröcks-del 1992^[42]^ | Primiparums tested positive for roll-over test between 28 and 32 weeks of gestation. | | Already manifest or preexistent hypertension impending premature delivery, preexisting disorders of the kidney, liver, stomach, heart or lungs, mental illness, substance abuse, diabetes mellitus, neoplastic illness, gastrointestinal disturbances with impaired medication resorption, abnormal ultrasound findings (dystrophy, fetal or uterine malformation, amniotic fluid anomalies), body weight exceeding 100kg, medication with anticoagulants or analgetics, or a known acetylsalicylic acid allergy. | | - |
| Sun 2020^[44]^ | Single fetus, normal fetal structure during intrauterine screening, and high risk factors for PE (at least one of the following) : history of chronic hypertension; No history of hypertension or renal disease, but high blood pressure 20 weeks before pregnancy, systolic blood pressure > 140mmHg or (and) diastolic blood pressure > 90mmHg, urinary protein negative; Previous history of PE during pregnancy. | | With diabetes, heart disease, hypertension, severe mental illness and chronic nephritis and other medical complications; The patient was accompanied by pregnancy complications such as placenta previa, placental abruption and threatened abortion. Allergic constitution; Twin or multiple pregnancies; Patients with myasthenia gravis. | | - |
| Talari 2014^[46]^ | Presence of a high-risk factor for PE, such as previous history of the disease, essential hypertension, positive family history or underlying vascular disorder, gestational diabetes mellitus, or maternal age < 20 years or > 40 years. | | Known history of salicylate allergy or present or past peptic ulcer, as well as cases with other medical disorders such as chronic renal disorders, thyroid diseases, and hepatic and cardiac disorders. | | PE was defined as development of hypertension (140/90 mmHg or more) plus proteinuria (>300 mg protein in the 24-hour urine sample). |
| Vainio 2002^[47]^ | Women at risk of PE or intrauterine growth retardation were recruited from the population of pregnant women routinely attending antenatal clinics in Tampere and its envirous. Anamnestic risk factors included a history of chronic hypertension, familial risk of PE (mother or sister), gestational diabetes, age <20 or >40 years, previous PE, previous intrauterine growth retardation, or previous intrauterine death.  The women were asked to attend for transvaginal Doppler ultrasound investigation at 12 to 14 weeks of gestation. In cases where a constant bilateral diastolic notch was found in the uterine arteries those concerned were asked to participate in a randomised pacebo-controlled trial. | | Gestational weeks <12 or >14, asthma, allergy to acetylsalicyclic acid, previous peptic ulcer, or the use of prostaglandin inhibitors within ten days before investigation. | | Pregnancy-induced hypertension was defined as a sustained blood pressure increase to levels of 140mmHg systolic or 90mmHg diastolic after 20 weeks of gestation. PE was defined as blood pressure changes as above and proteinuria (defined as >300mg/24 h or ≥1+ dipstick in a random urine sample). |
| Viinikka 1993^[48]^ | Arterial hypertension (blood pressure without treatment >140/90 mmHg already before pregnancy), or had had severe PE in previous pregnancy. None of them excreted protein (<300 mg/24 h) in urine before pregnancy. | | - | | - |
| Villa 2013^[49]^ | Gestation 12-13+6 weeks with at least one of the following risk factors: age < 20 or > 40 years, BMI > 30kg/m^2^, chronic hypertension, prior GDM or PE, small for gestational age, fetus mortus. | | Allergy to aspirin; tobacco smoking (during this pregnancy); multiple pregnancy; and a history of asthma, peptic ulcer, placental ablation, infammatory bowel diseases (Crohn’s disease, colitis ulcerosa), rheumatoid arthritis, haemophilia or thrombophilia (previous venous or pulmonary thrombosis or coagulation abnormality). | | PE (blood pressure ≥140 and/or 90 mmHg in two consecutive measurements and proteinuria > 0. 3 g/24 hours). |
| Wallenburg 1986^[50]^ | Judged to be at risk of PIH or PE because of an increased blood pressure response to intravenously infused angiotensin II. | | A history of hypertension or cardiovascular or renal disease; course of pregnancy in all cases had been uncomplicated, with a maximum diastolic blood pressure of 80 mmHg; taken drugs except oral iron supplements. | | PIH was defined as the presence of a diastolic blood pressure 95 mmHg on at least two occasions 6 or more hours apart. PIH and concomitant proteinuria (>0. 5 g/L) in the absence of a urinary tract infection was defined as PE. |
| Normal Women | |  |  | |  |
| Bakhti 2011^[16]^ | Primigest women consulting before the 10th week of amenorrhea without previous vasculo-renal pathology. | | Patients consulting later than the 10th week, patients with a counter-indication to the use of aspirin, patients with chronic arterial hypertension before pregnancy, patients with chronic nephropathy or with a known autoimmune disorder, twin pregnancies and diabetic women. | | PE defined by a systolic blood pressure >140 mmHg and diastolic blood pressure >90 mmHg with >300 mg per day proteinuria. |
| Golding 1998^[23]^ | All primiparae resident in the parishes of Kingston and St Andrew in Jamaica were eligible. | | Women were not permitted to enter the study after 32 weeks of gestation. | | Development of hypertension using any of the definitions: diastolic blood pressure ≥ 90 mmHg; systolic blood pressure ≥ 140 mmHg; rise of 25 mmHg in diastolic blood pressure; rise of 40 mmHg in systolic blood pressure. Proteinuric PE defined as any hypertension with proteinuria of ≥ l+. |
| Haapsamo 2010^[26]^ | Age <40 years, <4 previous ovarian stimulations and no contraindications for aspirin. | | - | | According to the criteria established by the Working Group, in pregnant women, hypertension is defined as a systolic blood pressure level of 140mmHg or higher or a diastolic blood pressure level of 90 mmHg or higher that occurs after 20 weeks of gestation in a woman with previously normal blood pressure. As many as one quarter of women with gestational hypertension will develop proteinuria, ie, PE. |
| Hauth 1993^[28]^ | Nulliparous, healthy, and with a singleton gestation at between 20 and 22 weeks' gestation. | | Illnesses or conditions known to increase the incidence of PE or pregnancy-induced hypertension, such as renal disease, collagen vascular disease, diabetes mellitus, multifetal gestation, and chronic hypertension. | | PE: a diastolic blood pressure ≥90 but < 110 mmHg on at least two occasions at least 1 hour apart and before or during labor or within 12 hours post partum and proteinuria of ≥1+ on two or more occasions at least 1 hour apart in the absence of a urinary tract infection or gross hematuria or ≥0. 5 g per 24 h. |
| Herabutya 1996^[29]^ | All normal nulliparous pregnant women conlined at Ramathibodi Hospital under the age of 30 years were selected after undergoing routine ultrasound at 18-22 weeks of gestation to confirm gestational age and to exclude anomalies. | | - | | - |
| Rotchell 1998^[40]^ | All women between 12 and 32 weeks of gestation without contraindications were eligible for entry into the trial. | | Increased risk of bleeding, known allergy to aspirin, high likelihood of immediate delivery or previous placental abruption. | | Initial diastolic blood pressure < 90 mmHg with an increase of ≥25 mmHg to ≥90 mmHg, or an initial pressure ≥90 mmHg with ≥15 mmHg increase, irrespective of antihypertensive agent use; with more than trace proteinuria. |
| Sibai 1993^[43]^ | Nulliparous women seeking prenatal care who were 13 to 25 weeks pregnant if their blood pressure was below 135/85 mmHg and they had no proteinuria on testing with a dipstick. | | Chronic hypertension, renal disease, diabetes mellitus, and other medical illnesses were excluded. | | PE was defined as hypertension (defined as a systolic blood pressure of ≥140 mmHg or a diastolic blood pressure of ≥90 mmHg) plus proteinuria (either ≥300mg per 24 hours or 2+ or more by dipstick on two or more occasions 4 hours apart) in the absence of a urinary tract infection. |
| Subtil 2003^[11]^ | Nulliparous (no previous delivery at or after 22 weeks), at a gestational age between 14 and 20^+6^ weeks, planned to continue prenatal care and give birth in the participating facility and provided written informed consent. | | Known history of hypertension, a potential indication (antiphospholipid antibodies, lupus) for or a contraindication (allergy, frequent haematomas or bleeding, history of haemorrhage during surgery, tooth extraction or other, recent gastric or duodenal ulcer, severe asthma) to aspirin or other anticoagulant treatment during this pregnancy. Multiple pregnancy was not an exclusion criterion. | | Pregnancy-related hypertension was diagnosed if, after 20 weeks of gestation, the patient’s systolic and diastolic blood pressure values equalled or exceeded 140 and 90 mmHg, respectively, in two successive measurements at least 4 hours apart. PE was diagnosed when pregnancy-related hypertension occurred together with permanent proteinuria, defined as at least two pluses (++) on the reagent strip or at least 0. 5 g/L in a urine test (unless measured during a urinary tract infection). |
| Taherian 2002^[45]^ | Nulliparity, single gestation, first prenatal visit before 20 weeks of gestation, systolic/diastolic blood pressure (BP) lower than 130/80 mmHg, and no proteinuria detectable by a dipstick. | | History of cardiovascular, renal or endocrinologic problems, medical or obstetric complications and those with known hazardous condition (multifetal gestation, hydatidi-form mole). | | Patients were considered to have mild PE if they demonstrated an increase of 30 mmHg in systolic or 15 mmHg in diastolic BP above the standard pressure. In addition, they should have demonstrated equal or greater than 300 mg/24 hours in urine collection, or in two random urine specimens obtained 4 hours apart and containing at least 1+ protein by the dipstickmethod. |
| Wang 1993^[51]^ | High risk of intrauterine growth retardation. | | - | | - |
