# Supplementary material for: Aspirin for the prevention of preeclampsia: A systematic review and meta-analysis of randomized controlled studies
Source: Front Cardiovasc Med. 2022 Nov 9;9:936560. doi: 10.3389/fcvm.2022.936560 (PMC9682183; doi:10.3389/fcvm.2022.936560)
Supplement: Supplementary file 3 [file Data_Sheet_3.pdf]

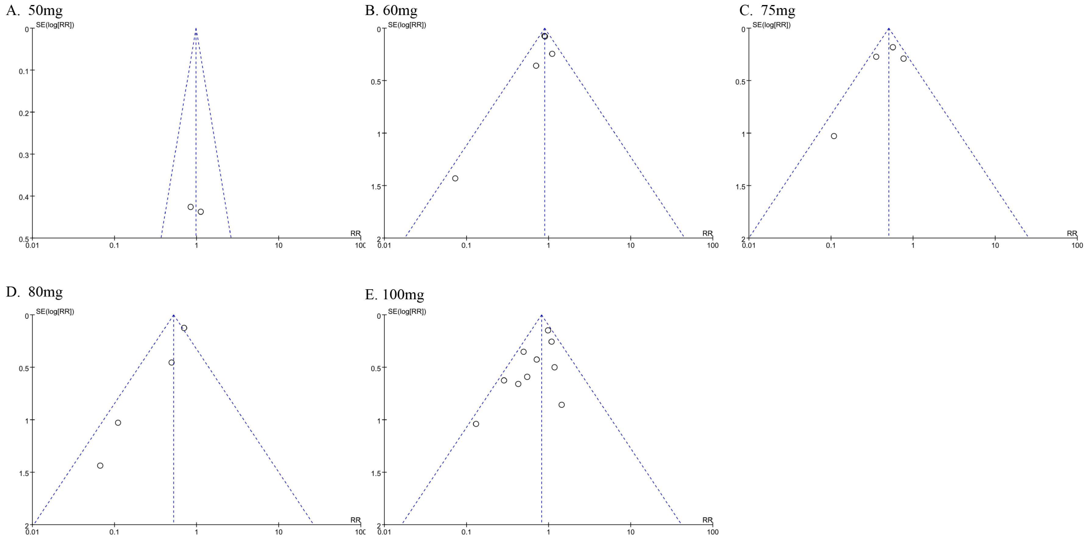

Figure 1 Funnel plot of subgroup analysis according to the dose of aspirin use. **(A)** 50 mg/day. **(B)** 60 mg/day. **(C)** 75 mg/day. **(D)** 80 mg/day. **(E)** 100 mg/day.

A. 60mg

Study	RR	95% CI
Omitting Bower 1996	0.89	0.80-0.99
Omitting Caritis 1998	0.88	0.77-1.01
Omitting CLASP 1994	0.89	0.77-1.04
Omitting ECPPA 1996	0.88	0.79-0.98
Omitting Wallenburg 1986	0.90	0.81-1.00
Total	0.89	0.80-0.99

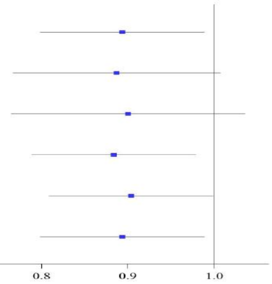

B. 75mg

Study	RR	95% CI
Omitting Byaruhanga 1998	0.42	0.24-0.74
Omitting Ebrashy 2005	0.43	0.20-0.94
Omitting McParland 1990	0.54	0.37-0.79
Omitting Sun 2020	0.58	0.35-0.97
Total	0.50	0.32-0.78

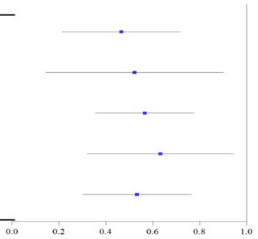

C. 80mg

Study	RR	95% CI
Omitting Abdi 2020	0.23	0.06-0.89
Omitting Movahed 2017	0.22	0.03-1.87
Omitting Schröcksnadel 1992	0.50	0.22-1.13
Omitting Talari 2014	0.53	0.24-1.18
Total	0.38	0.14-1.02

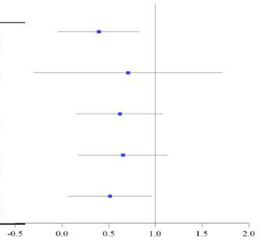

D. 100mg

Study	RR	95% CI
Omitting Ayala 2013	0.86	0.69-1.07
Omitting Grab 2000	0.81	0.66-0.99
Omitting Harrington 2000	0.80	0.65-0.99
Omitting Hermida 1997	0.83	0.68-1.02
Omitting Hermida 2003	0.77	0.62-0.97
Omitting Lin 2022	0.68	0.51-0.91
Omitting Liu 2016	0.85	0.69-1.04
Omitting Morris 1996	0.83	0.67-1.02
Omitting Schiff 1989	0.85	0.69-1.04
Omitting Villa 2013	0.82	0.67-1.02
Total	0.82	0.66-1.00

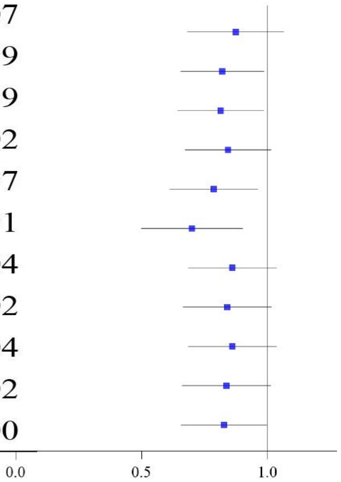

Figure 2 Sensitivity analysis of subgroup analysis based on aspirin dose. **(A)** 60 mg/day. **(B)** 75 mg/day. **(C)** 80 mg/day. **(D)** 100 mg/day.

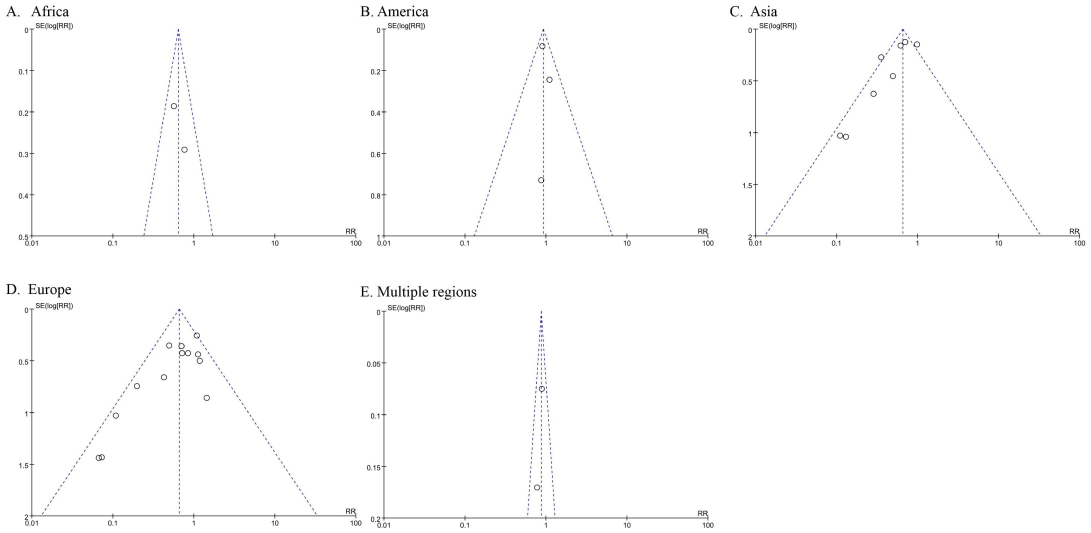

Figure 3 Funnel plot for subgroup analysis by region where the study was conducted. **(A)** Africa. **(B)** America. **(C)** Asia. **(D)** Europe. **(E)** Multiple regions.

A. America

Study	RR	95% CI
Omitting Caritis 1998	1.08	0.69-1.71
Omitting ECPPA 1996	0.90	0.77-1.06
Omitting Odibo 2015	0.92	0.79-1.08
Total	0.92	0.77-1.07

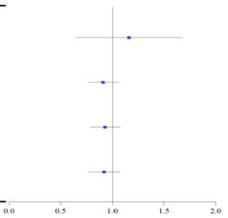

B. Asia

Study	RR	95% CI
Omitting Abdi 2020	0.65	0.54-0.78
Omitting Gu 2020	0.67	0.55-0.81
Omitting Lin 2022	0.52	0.43-0.63
Omitting Liu 2016	0.67	0.57-0.79
Omitting Movahed 2017	0.66	0.56-0.78
Omitting Schiff 1989	0.67	0.57-0.79
Omitting Sun 2020	0.70	0.59-0.83
Omitting Talari 2014	0.67	0.57-0.79
Total	0.65	0.56-0.77

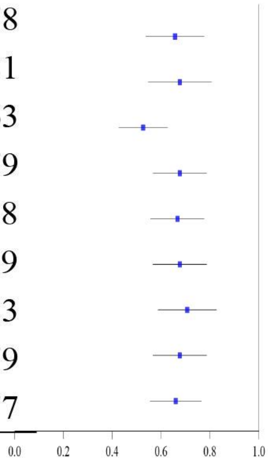

C. Europe

Study	RR	95% CI
Omitting Ayala 2013	0.69	0.53-0.89
Omitting Bower 1996	0.65	0.50-0.85
Omitting Grab 2000	0.64	0.50-0.83
Omitting Harrington 2000	0.63	0.49-0.81
Omitting Hermida 1997	0.67	0.52-0.86
Omitting Hermida 2003	0.57	0.43-0.75
Omitting McParland 1990	0.70	0.54-0.89
Omitting Parazzini 1993	0.62	0.48-0.80
Omitting Schröcksnadel 1992	0.69	0.53-0.88
Omitting Vainio 2002	0.69	0.54-0.89
Omitting Viinikka 1993	0.64	0.49-0.83
Omitting Villa 2013	0.65	0.50-0.84
Omitting Wallenburg 1986	0.69	0.54-0.88
Total	0.66	0.51-0.84

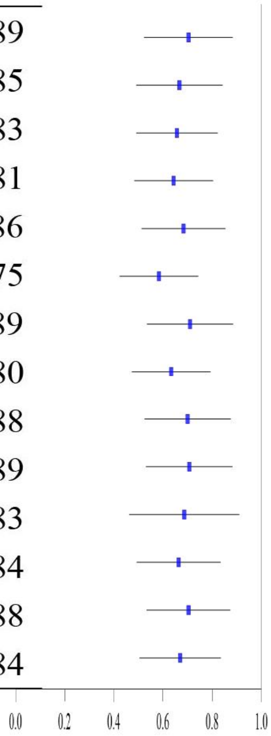

Figure 4 Sensitivity analysis for subgroup analysis by region where the study was conducted. **(A)** America. **(B)** Asia. **(C)** Europe.
